# New Caledonian crows attend to multiple functional properties of complex tools

**DOI:** 10.1098/rstb.2012.0415

**Published:** 2013-11-19

**Authors:** James J. H. St Clair, Christian Rutz

**Affiliations:** School of Biology, University of St Andrews, Sir Harold Mitchell Building, St Andrews KY16 9TH, UK

**Keywords:** animal tool use, comparative cognition, folk physics, hook, tool choice, tool selectivity

## Abstract

The ability to attend to the functional properties of foraging tools should affect energy-intake rates, fitness components and ultimately the evolutionary dynamics of tool-related behaviour. New Caledonian crows *Corvus moneduloides* use three distinct tool types for extractive foraging: non-hooked stick tools, hooked stick tools and tools cut from the barbed edges of *Pandanus* spp. leaves. The latter two types exhibit clear functional polarity, because of (respectively) a single terminal, crow-manufactured hook and natural barbs running along one edge of the leaf strip; in each case, the ‘hooks’ can only aid prey capture if the tool is oriented correctly by the crow during deployment. A previous experimental study of New Caledonian crows found that subjects paid little attention to the barbs of supplied (wide) pandanus tools, resulting in non-functional tool orientation during foraging. This result is puzzling, given the presumed fitness benefits of consistently orienting tools functionally in the wild. We investigated whether the lack of discrimination with respect to (wide) pandanus tool orientation also applies to hooked stick tools. We experimentally provided subjects with naturalistic replica tools in a range of orientations and found that all subjects used these tools correctly, regardless of how they had been presented. In a companion experiment, we explored the extent to which normally co-occurring tool features (terminal hook, curvature of the tool shaft and stripped bark at the hooked end) inform tool-orientation decisions, by forcing birds to deploy ‘unnatural’ tools, which exhibited these traits at opposite ends. Our subjects attended to at least two of the three tool features, although, as expected, the location of the hook was of paramount importance. We discuss these results in the context of earlier research and propose avenues for future work.

## 1. Introduction

The effective selection, modification or manufacture of tools requires some attendance to the physical properties of tool materials (such as mass, dimensions, shape or rigidity). Sensitivity to tool properties is likely to influence the efficiency (and profitability) of tool-oriented behaviours, and is thus highly relevant to the study of tool use as an ecological adaptation. New Caledonian crows *Corvus moneduloides* (henceforth ‘NC crows’ or ‘crows’) are notable among non-human tool users, both for the intricacy of their tool-manufacture techniques and for the dexterity of their tool-assisted extractive foraging. In the wild, NC crows produce several discrete tool types from a range of living and dead plant materials, including hooked stick tools crafted from branched twigs, and barb-edged pandanus tools removed from the margins of screw-pine (*Pandanus* spp.) leaves [1,2]. Despite such apparently sophisticated behaviour, the extent to which crows may pay attention to, let alone ‘understand’, the functional properties of their tools remains unresolved [3].

Several studies have explored aspects of tool selectivity in NC crows. Field efforts have shown that non-hooked stick tools used for extracting wood-boring beetle larvae are a non-random selection of the available raw materials [4] and that crows will replace tools that prove too short for a particular task with longer ones ([5], see also [6]). Laboratory studies with small numbers of wild-caught NC crows have demonstrated that at least some individuals are able to select straight sticks of an appropriate length [7], and diameter [8], to solve extraction tasks without the need for trial-and-error. Additionally, attendance to the polarity of tools with ‘functional’ and ‘non-functional’ ends has been shown in an experiment in which wild-caught subjects were presented with human-made stick tools that contained an awkward lateral extension that rendered one end of the tool unsuitable for probing with; all tested individuals preferred to use these tools in the functional (i.e. extension-upward) orientation, and usually ‘flipped’ such tools before using them if they had been experimentally provided in the non-functional (extension-downward) orientation [9].

NC crows are the only non-human animal species known to ‘craft’ hooked tools in the wild (although woodpecker finches *Cactospiza pallida* have recently been observed using naturally barbed blackberry twigs as tools [10]). Attendance to tool properties may be of particular importance when using hooked tool types, because it is in the nature of hooks to become non-functional when held in the wrong orientation. This said, the evidence that NC crows pay attention to hook functionality remains equivocal. A wild-caught captive bird that was presented with a choice between a straight and a hooked piece of wire for retrieving a baited bucket from a vertical tube (a task best accomplished with a hooked tool) appeared to choose tools randomly; on the other hand, when she did use the hooked wire she preferred to use it in its ‘hook-functional’ orientation [11], and when only straight pieces of wire were immediately within reach, she spontaneously bent them into hooked shapes ([11,12], see also [13]). In a follow-up experiment requiring the modification of aluminium strips, the same bird's performance was also inconsistent—she would often bend the material to form a hook, but tried to retrieve the bucket using the unmodified (‘wrong’) end of the tool in five of the first 10 trials [14]. Interestingly, a free-flying (wild) immature and an adult NC crow have been observed picking up their own recently manufactured hooked stick tools in the correct orientation with, respectively, little and no error [15]. By contrast, both free-flying and temporarily captive wild NC crows performed poorly in an experiment designed to test their attendance to the functional properties of ‘wide’ pandanus tools, rectangular leaf strips with barbs along one edge (two other pandanus tool designs have been described—for details, see [2,16]). When replicas of wide pandanus tools were presented in a non-functional orientation, inserted in a vertical hole with the barbs pointing downwards instead of upwards, subjects did not reverse the tools before use (thus making the barbs function as hooks), but generally used them, unsuccessfully, in the orientation in which they were first encountered [9]. Moreover, only a subset of birds eventually ‘flipped’ non-functional tools, correctly orienting the barbs. That the strategy of tool-flipping was adhered to when crows were having difficulty obtaining food with correctly oriented tools, and also when they were using experimentally modified wide pandanus tools that lacked any barbs whatsoever, strongly suggests that the crows paid scant attention to the barbed edge of the tools, let alone whether the barbs were in a functional orientation or not [9].

**Figure 1.**
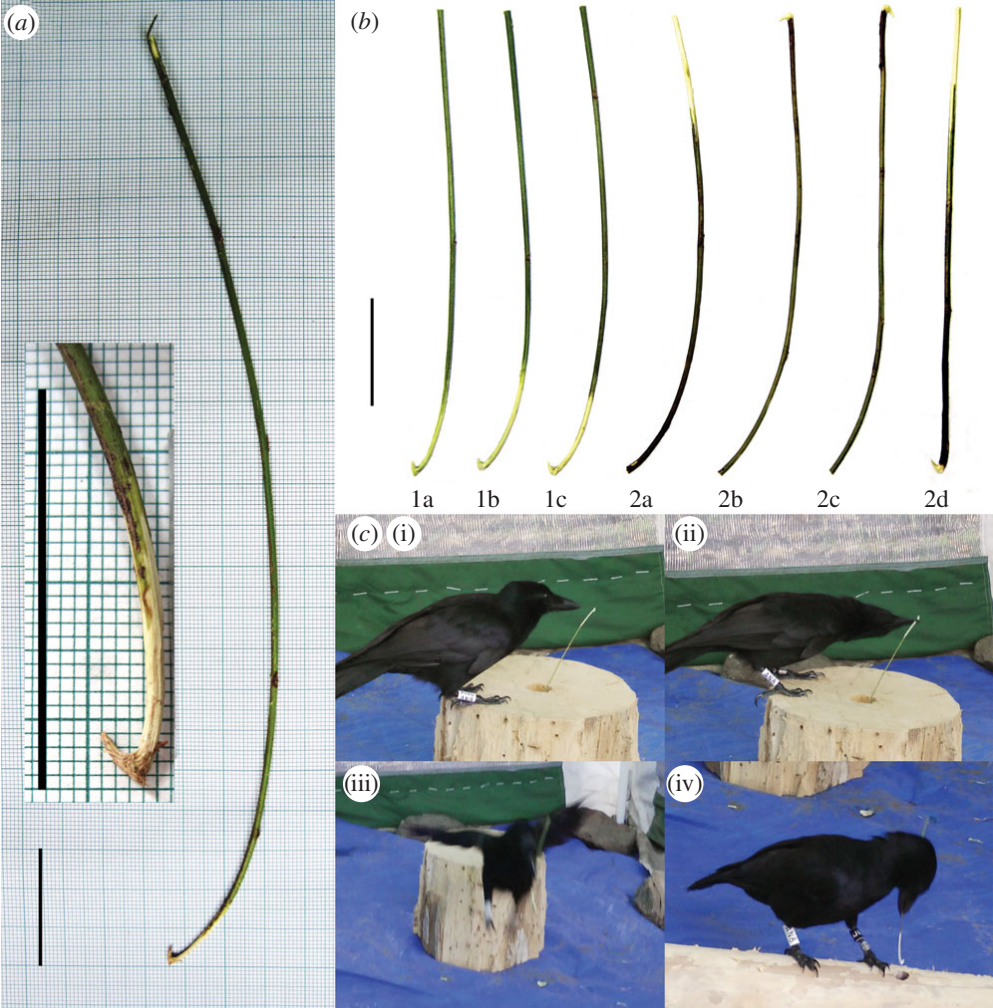
(*a*) A hooked stick tool manufactured by a New Caledonian crow from our Gouaro-Déva study site, showing the three co-occurring features investigated in experiment 2: a terminal hook; curvature of the tool shaft (greatest towards the hooked end) and a pale area of stripped bark (towards the hooked end). The inset shows (enlarged) the hooked portion of the same tool. Both scale bars, 3 cm. (*b*) Examples of a complete set of human-made replica tools corresponding to seven treatments across two experiments (codes match those in table 1); 1a–1c are naturalistic tools with all three features at the same end, whereas 2a–2d each have two features, one at each end of the tool, forcing binary choices. Scale bar, 3 cm. (*c*) Sequence of still images (from video) of a typical trial: (i) a crow on the tool-presentation log, about to pick up a tool in treatment 1b; (ii) crow picking up the tool; note that this individual, which prefers to position the non-working end of tools pressed against its left cheek, has entirely inverted its head in order to pick the tool up with the hooked end in its preferred ‘working position’, projecting to the right; (iii) crow transporting the tool, with the hooked end still in its preferred working position; and (iv) crow about to insert the tool into the baited hole in the food log. For further details, see text and table 1.

Such a result seems puzzling, given that unsuccessful tool deployment is likely to incur fitness costs in the wild [17,18]. To clarify whether a lack of attendance to tool functional properties during extractive foraging is a general feature of wild NC crows, we investigated whether subjects from a population that uses hooked stick tools attend to the key functional property of these tools, the hook. We did this by presenting replicas of hooked stick tools in a variety of orientations, and recording whether they were preferentially picked up and used in their hook-functional orientation. Hooked stick tools manufactured at our study site normally exhibit three co-occurring features which are located at the same end of the tool: a hook, marked curvature of the tool shaft and an area of stripped bark extending over a few centimetres of the tool shaft (figure 1*a*). As any of these features could potentially be used as criteria for orienting hooked tools correctly, we assessed their relative importance in a companion experiment, in which birds were provided with experimentally manipulated tools that forced binary choices between different features. This allowed us to investigate whether subjects were indeed paying attention to the hook or were instead basing their tool-orientation choices entirely on (normally) co-occurring features of different functional significance. This approach follows an established paradigm in animal tool-use research, in which animals are provided with experimentally manipulated tools in order to explore their appreciation of tool affordances [9,19–22].

Our experiments demonstrate that NC crows paid close attention to the functional properties of hooked stick tools, with no need for trial-and-error learning during the course of the experiment. We discuss explanations for the contrast between our results and those of the earlier study on wide pandanus tools [9] and place our findings in the context of what is known from other study systems. We also highlight profitable avenues for future research, such as exploring the possible functional significance of different tool features and uncovering the foraging contexts in which different pandanus and stick-tool types are used in nature.

## 2. Material and methods

### (a) Study site, subjects and husbandry

Between 20 October 2012 and 25 November 2012, NC crows were captured with meat-baited whoosh nets in our dry-forest study site (Gouaro-Déva) on the central-west coast of New Caledonia, South Pacific (for study site details see [4,17], and for a map see [23]). Birds were kept in temporary aviaries (3 × 3 m area; 2.5 m high at highest point). Subjects were housed individually, with the exception of adults captured with dependent young, which were co-housed to minimize stress (but separated for experimental trials). Housing aviaries were connected to experimental aviaries, in which all experimental trials were run (see below). Birds were fed a varied diet consisting of, among other items, meat, wet and dry cat food, rehydrated mealworms, pasta, nuts, vegetables and fruit, and had *ad libitum* access to water for drinking and bathing.

### (b) Pre-testing of subjects

Some aspects of NC crows' tool-oriented behaviour may be under the influence of social-transmission (‘cultural’) processes [16]. To address the ethical concern of exposing crows to a possibly unfamiliar tool type, we initially assayed the tool use of captured birds by providing a simple extraction task (holes drilled into a log, containing meat accessible only with tools) together with a sample of dry twigs and locally preferred raw materials for making hooked stick tools (branching stems of a non-native perennial). Of 15 birds pre-tested, six failed to manufacture hooked stick tools (including three dependent young) and one immature crow did not habituate well and was released before further testing. Accordingly, eight individuals progressed to the experiments detailed below (two adult males; one adult female and five immature females).

### (c) Scoring of subjects' laterality

Although scoring of how crows oriented supplied tools during food extraction was unambiguous (see below), we were also interested to learn whether crows might be making their choices much earlier, before the tool was deployed or perhaps even picked up. NC crows express strong lateral biases [24–27], preferring to hold their tools during use with the non-inserted part of the shaft pressed against either the left or the right cheek. Thus, prior to running any experimental trials, we assayed each subject's laterality by recording how it held its tools during nine successful extractions of food from baited holes. This enabled us to score, later on during experiments, which end of a given tool was held on the preferred non-working side, allowing us to infer that the opposite end of the tool was in the preferred ‘working position’ (figure 1*c*). This method was more useful than simply recording which end of the tool extended ‘in front of’ the bird, because it could be applied even in cases where a tool was held across, rather than parallel to, the bird's saggital plane.

### (d) Experimental set-up, rationale and tools

Experimental aviaries contained a central pole with 1–2 lateral perches and two pieces of experimental apparatus: an upright ‘tool-presentation log’ that was trimmed to be flat-topped and circular in cross section, forming a platform (approx. 35 cm high and 35 cm in diameter; figure 1*c*(i)) on which tool presentations were made (for further details, see below); and a ‘food log’, positioned *ca* 1 m from the tool-presentation log, containing a single drilled hole (7 cm deep and 1.6 cm in diameter) from which a peanut-sized piece of beef or pork heart could be extracted with supplied tools (figure 1*c*(iv)). No food or potential tools were present other than those provided as part of the experiment, although water was always available *ad libitum*. The mesh side walls of experimental aviaries were covered with semi-opaque screening, to reduce distraction to the subject and to ensure that crows in nearby housing aviaries, and wild birds, could not observe experimental trials. Subjects took part in two experiments, consisting of three and four tool presentations, respectively (‘treatments’; for details, see below and table 1).

**Table 1.** Summary of treatments and key results from two experiments investigating the attendance of New Caledonian crows to the features of hooked stick tools. (In the schematic section, parts of the tool shaft stripped of bark are shown in light green, whereas unstripped parts are dark green (cf*.* figure 1*a*). The bottom three rows summarize results for tool-orientation choices at, respectively, the pick-up, transport and use stages of the treatment in question (results for the use stage given in bold font). Each result cell identifies a feature at one end of the tool and (in brackets) the number of crows out of the total sample that chose this feature instead of the alternative. Finally, two-tailed probabilities for binomial tests are given.)

	Experiment 1			Experiment 2			
treatment	a	b	c	a	b	c	d
tool type	‘naturalistic’	‘naturalistic’	‘naturalistic’	curve versus strip; no hook	curve versus *cis* hook; no strip	curve versus *trans* hook; no strip	hook versus strip; no curve
presentation	flat	hooked end upward	hooked end downward	flat	flat	flat	flat
tool schematic	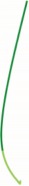	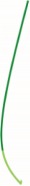	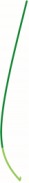	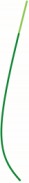	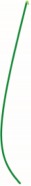	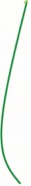	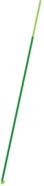
feature in working position at pick-up	hook (6/8; *p* = 0.289)	hook (5/8; *p* = 0.727)	hook (8/8; *p* = 0.008)	curve (4/8; *p* = 1.000)	hook (2/7; *p* = 0.453)	hook (4/8; *p* = 1.000)	hook (3/8; *p* = 0.727)
feature in working position during transport	hook (7/8; *p* = 0.070)	hook (6/7; *p* = 0.125)	hook (8/8; *p* = 0.008)	curve (7/8; *p* = 0.070)	hook (5/7; *p* = 0.453)	hook (6/8; *p* = 0.289)	hook (8/8; *p* = 0.008)
**feature inserted during use**	**hook (8/8; *p* = 0.008)**	**hook (7/7; *p* = 0.016)**	**hook (7/7; *p* = 0.016)**	**curve (8/8; *p* = 0.008)**	**hook (6/7; *p* = 0.125)**	**hook (7/8; *p* = 0.070)**	**hook (8/8; *p* = 0.008)**

The first experiment (‘experiment 1’) was designed to determine whether crows recognize which end of a normal hooked stick tool should be oriented towards prey in order for the tool to function as a hook. Because the context in which a tool is encountered may affect a crow's decision of how to orient it for use (see Discussion), we presented tools in three different orientations: lying flat on a surface (treatment 1a); inserted into a shallow hole with the hooked end pointing upward (treatment 1b) and inserted into a shallow hole with the hooked end pointing downward (treatment 1c; for further details, see below).

As described above, crow-made hooked stick tools at our study site typically exhibit three features which co-occur at the same end of the tool shaft (hook, curvature and stripped bark; figure 1*a*). Because any of these features could be used by crows to inform their choice of how to orient hooked stick tools correctly, we conducted a companion experiment (‘experiment 2’) that investigated their relative importance to crows as criteria for making tool-orientation decisions. To achieve this, we presented each subject with a series of tools that each lacked one of the three usually co-occurring traits, and in which the remaining two features were present at *opposite* ends of the tool shaft.

All tools used in the study were human-made, from fresh, locally sourced stems of the preferred plant material. In experiment 1, we manufactured replicas of crows' hooked stick tools, based on a sample of tools recovered from wild birds in our study site and those manufactured by subjects during pre-testing trials (see above). In experiment 2, tools were experimentally manipulated, by removal (or non-removal) of hooks, stripping (or non-stripping) of bark and straightening (or recurvature) of the tool shaft (by binding to a wire template and steaming for 10 min; see table 1 for a summary of treatments, figure 1*a* for a crow-made tool and figure 1*b* for human-made experimental tools). The raw materials used to make all tools presented to a given subject were first matched for diameter and length, and then randomly allocated to experiment 1 or 2, and to treatments within experiments. As every subject received a unique set of tools, we avoided pseudo-replicating experimental stimuli [28], thus allowing general inferences about crows' attendance to tool features.

In treatments 1a and 2a–2d, each tool was presented lying flat on top of the tool-presentation log, with its compass orientation determined at random. In treatments 1b and 1c, each tool was inserted into a wide, shallow hole (*ca* 3 cm deep and 2.5 cm in diameter) drilled into the centre of the tool-presentation log (see above), such that its shaft projected at approximately 25° from vertical and both of its ends were clearly visible to a crow standing on top of the tool-presentation log (figure 1*c*(i)).

Experiment 1 always preceded experiment 2, although within experiments, the order of treatments was randomized. In most cases, all seven tool presentations were made consecutively, but some individuals lost motivation after several food rewards had been obtained, and the remaining treatments were postponed until the following day.

### (e) Data collection and analysis

Once a crow had entered the experimental aviary (through a door connecting housing and experimental aviaries; see above), an assistant placed a single tool on the tool-presentation log, baited the food log and left the aviary. An observer filmed the crow's subsequent actions from a hide at one side of the experimental aviary. Once the crow had picked up the provided tool, transported it to the food log and attempted to extract the meat (usually successfully), the observer used a radio to notify the assistant that the treatment was complete. The assistant then re-entered the aviary, collected the old tool, provided the appropriate tool for the following treatment and rebaited the food log. This procedure was repeated until each crow had experienced each experimental treatment, with the exception of one subject which did not experience treatment 2b owing to an oversight.

From video, we subsequently recorded which end of each tool was inserted into the food log at first use (figure 1*c*(iv)). We also recorded, at the moments of picking up (figure 1*c*(ii)) and transporting (figure 1*c*(iii)) each tool, which end of the tool was positioned in the individual's preferred working position (for details on scoring lateral preferences, see above). Accordingly, during each treatment, each individual yielded a single tool-orientation datum at each of three time points—pick-up, transport and first use. All videos were analysed by the same observer (J.S.C.), with replicate/blind scoring considered unnecessary given the unambiguous nature of the behaviours of interest. Choice data from each experimental treatment were analysed using binomial tests, as each subject chose one of the two ends of the supplied tool to hold in the working position during pick-up and transport, and to insert into the food log during use. Two-tailed probabilities are reported throughout.

## 3. Results

### (a) Laterality assay

Seven out of eight birds expressed significant lateral biases, holding tools against the same cheek in all nine extractions (three preferred to position the non-working end of the tool on their left side and four preferred their right; all *p* = 0.004). One bird exhibited weaker laterality, preferring its right side in seven cases and the left side in two; although statistically non-significant (*p* = 0.18), for the purposes of this study, we treated this subject as preferring to position the non-working end on the right side.

### (b) Experiment 1: ‘naturalistic’ tools

**Figure 2.**
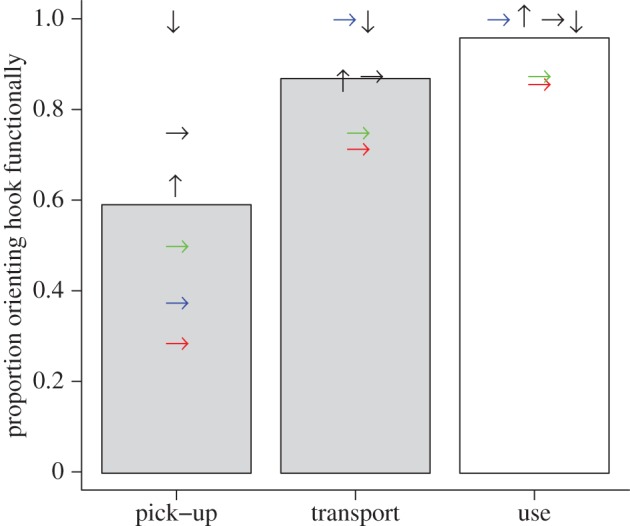
Proportion of New Caledonian crows in each treatment that held the hooked end of the tool in the working position during three different tool-handling stages: picking up, transporting and using the tool to extract meat from a hole (for scoring details, see Methods). The orientation of symbols indicates the orientation in which tools were presented to crows during trials (→, horizontal; ↑, hooked end upward; ↓, hooked end downward). Black symbols summarize trials with naturalistic replica tools (figure 1*b*) where the choice was between the end containing all co-occurring features, and the end containing none (treatments 1a–1c), whereas coloured symbols summarize trials with experimental tools which forced binary choices between specific tool features: green (treatment 2b) and red (treatment 2c) symbols indicate trials where the choice was between hook and curvature, and blue indicates trials where the choice was between hook and stripped bark (treatment 2d); trials with a choice between tool-shaft curvature and stripped bark (treatment 2a) did not contribute data relevant to this plot. Symbols are jittered where necessary to avoid overlap, and sample sizes are provided in table 1. Bars show the average of all points in each handling stage and are coloured according to how tool orientation was scored (grey bars, scored based on subjects' laterality; open bar, scored according to which end of the tool subjects actually inserted to probe for food).

All subjects used the hooked end of the tool for probing from the first attempt in all three treatments (table 1 and figure 2); note that one subject was excluded from treatments 1b and 1c because it broke off the hooks prior to use, possibly in an attempt to adjust shaft curvature (after pick-up in 1b and after transport in 1c). In most (treatments 1a and 1b) or all (treatment 1c) cases, naturalistic tools were also picked up and transported with the hooked end positioned in the preferred working position (table 1 and figure 2).

### (c) Experiment 2: ‘unnatural’ tools

In treatment 2a, all eight subjects used the curved-shaft end of the tool (rather than the stripped-bark end) for probing from the first attempt (table 1 and figure 2). In treatments 2b and 2c, all but one subject probed with the hooked end of the tool, rather than the curved end (the same individual chose the curved end in both treatments), and in treatment 2d, all subjects probed with the hooked rather than the stripped-bark end (table 1 and figure 2). Note that one bird was not run on treatment 2b owing to an oversight (see Methods).

## 4. Discussion

In our experiments with wild-caught, hooked stick-tool making NC crows, we found: (i) that subjects paid close attention to which end of human-made naturalistic replica tools were hooked; (ii) that they deployed these tools in hook-functional orientation regardless of how they had been presented initially; and (iii) that in most cases, the preference for inserting hooks quickly overrode information from (usually) co-occurring characteristics of hooked stick tools, namely tool-shaft curvature and an area of stripped bark.

The finding that our subjects were sensitive to the properties of hooked stick tools contrasts with results from an earlier study (see Introduction), in which birds appeared to pay little or no attention to the barbs on supplied wide pandanus tools, often using them in the wrong (barb-non-functional) orientation [9]. To eliminate any conflict between potentially competing social (tool insertion) and non-social (barb orientation) sources of information, a more thorough test would have included the presentation of tools in a neutral (i.e. not pre-inserted) orientation, as in treatment 1a of our study. Nevertheless, the results of the earlier experiment [9] contrast with the findings from our (comparable) treatments 1b and 1c, strongly suggesting that NC crows' attendance to the key functional feature of wide pandanus tools is relatively weak. Given that both tool types are functionally highly polarized (i.e. they must be inserted in a specific orientation for the hook/barbs to function effectively) and that the fitness costs of incorrect deployment are likely to be similar, the observed differential attendance to functional tool features begs an explanation.

It has been proposed that NC crows can afford to ignore the functional features of pandanus tools during deployment because the manufacturing process—the sequence of bill-made cuts and rips that detach the tool from the living leaf—is so stereotyped within individuals that birds almost invariably end up holding their tools in the correct orientation immediately after manufacture [9,29]. In the case of hooked stick tools, we can confidently reject such ‘procedural knowledge about the sequence of [manufacturing] operations’ [9, p. 1] as an essential mechanism for correct orientation during use; despite these tools' suggested stereotyped manufacture [15], all subjects in our experiment 1 were able to use non-self-manufactured hooked stick tools in the correct orientation at the first attempt. We therefore conclude that NC crows employ different strategies for correctly orienting these two tool types, which suggests that attendance to their functional features is subject to different constraints. These may be ontogenetic in nature, as for example, attendance to some types of tool trait may be more easily acquired than others. A protracted juvenile development period is often necessary in order to learn particular tool-related skills [4,30], an idea that is supported by empirical data for wild NC crows [4,31,32]. The adoption of ‘fast and frugal’ heuristics [33,34] may serve to accelerate this costly life-history stage (and to reduce associated fitness costs, including those arising from delayed reproduction), but such short-cuts may not be equally feasible for all tool types: the barbs on pandanus leaves are a pre-existing part of the material and NC crows could certainly learn to manufacture functional pandanus tools without ever attending to this feature [31], whereas a failure to learn to attend to the hooked end of hooked stick tools would necessarily go hand in hand with failure to develop the ‘crafting’ behaviour that is central to their manufacture [15].

Apart from ontogenetic constraints, there may be a direct cost of attending to tool features, such as a small time penalty or increase in ‘cognitive load’ each time a tool-orientation decision is made. The magnitude of any such cost may correlate with the physical conspicuousness of the functional feature(s) in question. Specifically, hooked stick tools contain several highly conspicuous features which may facilitate fast and reliable identification of the functional (hooked) end, while achieving the same result with wide pandanus tools would require attendance to a single relatively subtle feature, the directionality of small barbs [9]. Consistent with the idea that marked phenotypic polarity facilitates attendance to functional polarity, it has been shown that NC crows provided with non-hooked stick tools with an awkward lateral extension at one end (wrongly identified as ‘hooked stick tools’ in a recent review [34]), generally chose to insert the end without the extension [9].

We found that our subjects could discriminate between three distinct tool features (each of which contributes to the phenotypic polarity of the tool) and that these features affect tool-orientation decisions differentially. In fact, our subjects' choices followed a remarkably consistent hierarchy, with the hook being generally preferred to tool-shaft curvature, which was in turn preferred to stripped bark. We note that a preference for curvature over the hooked end by a single subject, coupled with our modest sample size, led to non-significant results for treatments 2b and 2c, in which (respectively) six of seven and seven of eight subjects used the hooked end. An additional treatment would be required to determine whether stripped bark is preferred to anything at all, although our results suggest that it is used as a cue for initial tool-orientation decisions (4 out of 8 and 5 out of 8 crows picked up tools with the stripped end in the working position in treatments 2a and 2d, respectively, but all of these reversed tool orientation prior to use). While the ability of NC crows to identify different features within the same tool in a consistent way does not imply an understanding of their relative functional importance, we suggest that such discrimination is a prerequisite for the selective modification of functional features within a given tool (see also [35]), and may increase the potential for technological development. We hypothesize that the probability of successful innovation is greater in modular technologies, just as organismal modularity may enhance adaptive evolution [36,37].

We not only examined which end of each provided tool was actually used to extract food, but we also recorded which end was held in the individual's preferred working position at two earlier time points, namely when picking up and transporting the tool. We found that the majority of our subjects picked up naturalistic, non-manipulated tools with the hooked end in the working position (experiment 1; black symbols in ‘pick-up’ column of figure 2), but conversely, when experimentally manipulated tools were presented in order to force a binary choice between different tool features, half or fewer than half of the subjects picked them up with the hooked option in the working position (treatments 2b–2d; coloured symbols in ‘pick-up’ column of figure 2). Most of the remaining birds, however, subsequently re-oriented their tools so that the hooked end was in the working position prior to transport and use, demonstrating that they had paid attention to the hook. Although our interpretation inevitably remains tentative owing to small sample sizes, the observed pattern suggests that both tool-shaft curvature and stripped bark were used as cues for initial tool orientation and that additional information (hook location) was subsequently used to correct unsatisfactory initial decisions. To human eyes at least, tool curvature and stripped bark are more evident morphological features than hooks (being on the scale of centimetres rather than millimetres; figure 1*a*), and so it is conceivable that NC crows use the gross phenotypic polarity of their hooked stick tools as an initial criterion for tool orientation before paying attention to the hook.

The ability to discriminate the functionality of alternative tools in experimental choice tests [19,38,39] is conceptually similar to the ability, described here, to discriminate which end of an individual tool contains the functional features. Studies of tool selectivity often have the stated aim of determining whether subjects ‘understand’ the functional properties of their tools (reviews: [34,40]), although we suspect that this is seldom, if ever, achieved. In any case, we have little to contribute to this particular debate because our experiment was simply designed to determine whether NC crows attend to the features of hooked stick tools, and to explore the relative importance of these features as criteria for tool orientation. Our subjects' striking preference to work with the hooked end of tools is *not* evidence for a causal understanding of how hooks function (whatever a ‘causal understanding’ may be [3])—the observed preference could feasibly be owing to an (evolved) neurological predisposition or to ontogenetic (learning) processes (see above), or most likely to a combination of both mechanisms. For perspective, few would argue that a hermit crab has a causal understanding of the functionality of mollusc shells with different properties, but this does not preclude their making functional choices [41].

Irrespective of the cognition underlying crows' tool-orientation decisions, it is possible that all three investigated features of hooked stick tools, individually or in combination, improve the performance of the tool during foraging. It seems reasonable to assume the functionality of the hook, so this is not discussed further here. Tool-shaft curvature may serve to ensure that the tool tip can be positioned in the centre of the crow's field of binocular vision when the tool is held in the preferred transverse grip [27]. Curvature may thus substantially improve the accuracy and/or precision with which the tool tip can be positioned in space. Next, although it is possible that the stripping of bark from the tool shaft at the hooked end is a ‘spandrel’ [42] resulting from the crafting of the hook and removal of loose fibres, at the risk of seeming Panglossian we can envisage two possible functions. First, the exposure of smooth woody material may reduce friction against the sides of holes and crevices, increasing the energy efficiency of probing and making the tool more likely to slide past or through the bodies of prey animals. Second, the removal of relatively dark green or brown material exposes the much brighter wood beneath (figure 1*a*), providing maximum contrast, and thus perhaps improving visibility when the tool is deployed in low light conditions. While conjectural at present, the possible effects of each of the features on hooked stick tool functionality are accessible to experimentation—a route we are productively pursuing.

Related to the previous point, it would be interesting to know whether the order in which our subjects preferred the different tool traits matches their relative contributions to tool efficiency. If the hook contributes most to the tool's efficiency, then it could be argued that the crows' preference for hooks is consistent with an appreciation of hook functionality (where ‘appreciation’ is defined as the recognition of value, without implying particular cognitive processes such as causal reasoning). We suggest that future studies of the functional properties of tools should go hand in hand with investigations of the ecological context of tool deployment, as very little is currently known about the foraging function of most NC crow tool types/designs [2]. Non-hooked (‘straight’) stick tools, such as those used for extracting large longhorn beetle larvae from their burrows in dead wood (so-called ‘larva-fishing’), would provide an interesting comparison in future experiments on the specificity of tool functions, as these are usually pieces of dead plant material that exhibit no obvious crow-induced curvature or bark-stripped sections [4].

In conclusion, our results demonstrate that hooked stick-tool making NC crows pay attention to the functional characteristics of their tools, allowing them to orient tools correctly without relying on trial-and-error, on circumstantial evidence (such as tool orientation upon discovery) or on remembering the exact placement of tools when they were last put down. This close attendance to functional features demonstrates that the simple trial-and-error heuristic apparently used to orient wide pandanus tools is unlikely to reflect a species-wide incapacity; rather, it suggests that NC crows apply distinct strategies to the deployment of different tool types. The ability to recognize the functional orientation of tools has implications for the timescales over which tools may be profitably curated—individuals may re-use tools effectively whether or not they recall the orientation in which they were last put down—and for the frequency and profitability of adopting tools discarded by others, which is potentially a key mechanism for the social learning [4] and diffusion [43] of tool-related information in crow populations. Finally, the ability of NC crows to distinguish between, and even selectively modify, different functional features within a single tool may affect the evolution—cultural and/or genetic—of tool complexity [36].

## Notes

All experiments were approved by Oxford University's local ethical review committee, for the duration of the research grant that funded the work (this grant has recently been transferred from the University of Oxford to the University of St Andrews).
